# Expansion in speech time can restore comprehension in a simultaneously speaking bilingual robot

**DOI:** 10.3389/frobt.2022.1032811

**Published:** 2023-03-01

**Authors:** Hamed Pourfannan, Hamed Mahzoon, Yuichiro Yoshikawa, Hiroshi Ishiguro

**Affiliations:** ^1^ Intelligent Robotics Laboratory (Hiroshi Ishiguro’s Laboratory), Department of Systems Innovation, Graduate School of Engineering Science, Osaka University, Osaka, Japan; ^2^ Institute for Open and Transdisciplinary Research Initiatives (OTRI), Osaka University, Osaka, Japan

**Keywords:** bilingual robot, competing-talker speech, human-robot interaction, pause duration, speech comprehension, speech expansion, user experience

## Abstract

**Introduction:** In this study, the development of a social robot, capable of giving speech simultaneously in more than one language was in mind. However, the negative effect of background noise on speech comprehension is well-documented in previous works. This deteriorating effect is more highlighted when the background noise has speech-like properties. Hence, the presence of speech as the background noise in a simultaneously speaking bilingual robot can be fatal for the speech comprehension of each person listening to the robot.

**Methods:** To improve speech comprehension and consequently, user experience in the intended bilingual robot, the effect of time expansion on speech comprehension in a multi-talker speech scenario was investigated. Sentence recognition, speech comprehension, and subjective evaluation tasks were implemented in the study.

**Results:** The obtained results suggest that a reduced speech rate, leading to an expansion in the speech time, in addition to increased pause duration in both the target and background speeches can lead to statistically significant improvement in both sentence recognition, and speech comprehension of participants. More interestingly, participants got a higher score in the time-expanded multi-talker speech than in the standard-speed single-talker speech in the speech comprehension and, in the sentence recognition task. However, this positive effect could not be attributed merely to the time expansion, as we could not repeat the same positive effect in a time-expanded single-talker speech.

**Discussion:** The results obtained in this study suggest a facilitating effect of the presence of the background speech in a simultaneously speaking bilingual robot provided that both languages are presented in a time-expanded manner. The implications of such a simultaneously speaking robot are discussed.

## Introduction

The world has never been as interconnected as it is today (Steger, 2017). Thanks to the advance in fast and reliable international transportation, borders are losing their value both from an economic and cultural perspective (Mohanty et al., 2018). At any given moment, around half a million people are in the air, traveling from one place to another (Spike, 2017). This provides a great opportunity for people of different countries, and language backgrounds to communicate, interact, and share their thoughts and ideas in international social spaces. Such social spaces can include exhibitions, airports, conferences, Expo events, museums, amusement parks, etc. Social robots have already been introduced to such social spaces around the world (Mubin et al., 2018). From food recommender robots to museum guides, and teaching assistants, to customer engagement and hotel receptionists (Herse et al., 2018; Duchetto et al., 2019; Yoshino and Zhang, 2020; Holthaus and Wachsmuth, 2014).

Considering the necessity of having social robots capable of serving people in their own preferred language, attempts have been made in the past to create social robots that can speak more than one language. Translator robots to help tourists in Japan (Nova, 2015), Tokyo Olympic guide robots (Srivastava, 2017), and Mitsubishi receptionist robot Wakamaru (Hanson and Bar-Cohen, 2009) are all examples of such an attempt to optimize robots for international settings. All the currently existing multilingual social robots, however, work in a one-to-one manner. Meaning that although some of them can speak more than 5 different languages (Xu et al., 2020), they can speak each of the languages in a different session. For instance, if the robot in hand can speak both English and Spanish, each of the clients should wait for the other person’s conversation with the robot to finish before they start a new talk in their preferred language. Despite the noticeable cost-efficiency of the currently existing multilingual robots considering the high cost of having several multilingual attendants to help people in international gatherings, the current situation can be further improved.

**FIGURE 1:**
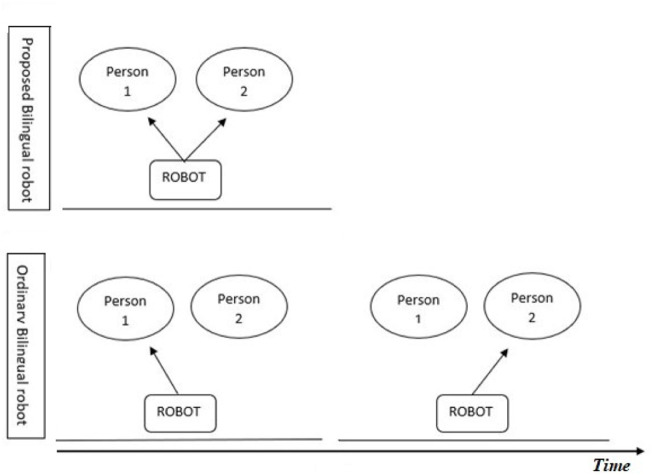
Depiction of how a simultaneously speaking bilingual robot **(A)** is more time-efficient than a conventional bilingual robot **(B)**.

By making multilingual social robots capable of speaking with more than one client at the same time, which we hereby refer to as the Simultaneously speaking Bilingual Robot, we can increase the time efficiency of such robots significantly. The above-mentioned scenario is depicted in Figure 1.However, one of the basic problems to be tackled in designing such a Simultaneously speaking Bilingual Robot is the well-documented deteriorating effect of background noise on speech comprehension. A large body of evidence exists supporting the negative effect of different types of background noise on the comprehension and retrieval of the presented auditory information. The investigated noises range from white noise (Jafari et al., 2019) to instrumental and vocal music (Smith, 1985), babble noise, and human speech (Salamé and Baddeley, 1982; Tremblay et al., 2000; Brännström et al., 2018). This negative effect is more enhanced when using human speech as background noise, also referred to as a competing talker scenario. This observed adverse effect is suggested to be due to our brain’s special sensitivity to the spectral properties of human speech (Albouy et al., 2020).

One way to take care of the negative effect of background noise on speech comprehension in a competing talker scenario is the adjustment and optimization of paralinguistic factors. Paralinguistics refers to the study of vocal cues that can facilitate communication of meaning through non-lexical means (Lewis, 1998). Such Paralinguistic factors include voice gender, fundamental frequency (pitch) of voice, speech rate, pauses during the speech, and intonations (Sue and sue, 2016). In a previous study, the effect of the robot’s voice gender and voice pitch on the speech comprehension of subjects listening to a simultaneously speaking bilingual robot has been investigated by the authors (Pourfannan et al., 2022). In the current study, the effect of speech rate and pause duration on the speech comprehension of subjects listening to a simultaneously speaking bilingual robot is in mind.

In general, the nature of the relationship between speech comprehension and the rate of speech is relatively clear (Weinstein-Shr and Griffiths, 1992). A slower speech rate is suggested to be easier to follow and understand (Picheny et al., 1986). A fast speech rate has been shown to harm speech processing if it exceeds a certain limit (around 400 words per minute) and is almost unintelligible when over 1,200 wpm (Du et al., 2014). When considering the subjective evaluation of subjects into account as well, things get more complicated as a slower speech rate can reduce the positive evaluation of subjects about the speaker by rating it more “passive,” and less trustworthy (Apple et al., 1979). Previous research investigating speech rate in the human-robot conversation suggests that the trend seems different when dealing with robot speech. While in human-human interaction, slow speech is usually rated lower, in human-robot interaction, moderately slow speech results in higher comprehension and subjective evaluation of subjects in comparison with faster speech rates (Shimada and Kanda, 2012).

To change the speed of speech, however, is not the only way to change the impression of subjects about the speech rate. Previous works suggest that adding to the duration, and frequency of pauses inside the speech can change the subjective evaluation of participants on the speed of speech without changing the actual speech rate (Liu et al., 2022). It is suggested that increased pause duration results in a perceived decrease in the speech rate in the subjects. The pause duration of o.6 s within the sentences and 0.6–1.2 s between the sentences is suggested to result in the highest naturalness of speech (Lin et al., 2021). In a clever work by Tanaka et al., it was shown that speech comprehension did significantly improve by a relatively short expansion in the speech time (as short as 100 m) if there were long enough pauses (300–400 m) between the phrases of the speech (Tanaka et al., 2011). They showed that both younger and older adults experienced a boost in their speech comprehension in noisy conditions when both the speech and pause duration were expanded. Furthermore, the same work suggests that the best performance is obtained when the effect of pause duration expansion and speech expansion is combined. As they put it this way “Intelligibility in sentences with 200 m pause and 200 m expansion was higher than those with 400 m pause and 0 m expansion”.

This is not the only work that shows the positive effect of expansion in speech time and pause duration on speech comprehension in noise. In another study, researchers have shown how by using a method called the “Clear Speech Technique” they managed to increase the speech comprehension of older adults listening to a challenging speech in noise (DiDonato and Surprenant, 2015). Clear speech is defined as a kind of speech that is spoken by a speaker who regards his/her audience as individuals with a hearing impairment or non-native to the spoken language (Ferguson and Kewley-Port, 2007). In this work, they show that expansion in the speech time and pause duration in addition to other acoustic modifications (e.g., increased size and duration of vowels) can turn an otherwise challenging listening task into a “relatively effortless” one.

### Research rationale

Considering the large body of evidence in hand, we know that speech comprehension tends to deteriorate in noisy conditions and this effect is stronger in multi-talker situations when the background noise is human speech, even if the individual does not understand that language (Klatte et al., 2007). Speech rate and pause duration as two main paralinguistic factors have been shown to facilitate speech comprehension in noise, and their accumulative effect is higher than the effect of each one of them on its own. A model of presenting the speech material is suggested based on previous works that promise to increase speech comprehension using an expansion in the speech time and pauses duration in the speech. The current work applies this method to increase speech comprehension in a bilingual competing talker scenario where the aim is to boost the understanding of both parties when listening to the robot’s speech in their own language at the same time.

With this purpose in mind, we designed a set of four experiments. We compared standard-speed monolingual speech with the standard-speed bilingual speech in the first experiment. The hypothesis was that the score of subjects in the monolingual condition will be higher than the bilingual condition. The reason behind this hypothesis was the large body of evidence in support of the negative effect of background speech on the comprehension of the target speech in a dual-talker speech scenario. We compared standard bilingual speech with the expanded bilingual speech in the second experiment. The hypothesis was that the expanded bilingual speech will outperform the standard speed bilingual condition. This would be in accordance with previous studies on the positive effect of speech expansion on speech comprehension in noisy conditions. In the third experiment, we compared standard-speed monolingual speech with expanded bilingual speech. It was hypothesized that the relative effort imposed by the presence of background speech, in addition to the extra cognitive resources introduced by the expansion of speech may in fact be able to improve the performance of subjects in an expanded bilingual speech in comparison with the standard monolingual speech. And, in the fourth experiment, the expanded monolingual speech was compared with the expanded bilingual speech. It was hypothesized that the extra cognitive resources provided by speech expansion, in addition to a relative amount of effort imposed by the presence of another expanded language in the background may be able to outperform the expanded monolingual condition where there is no extra effort required to trigger the utilization of the extra cognitive resources provided for the participant.

## Material and Methods

### Participants

A total of 182 participants between the age of 20–40 years old (M = 32, SD = 6.5) were recruited using the Prolific online research participant requirement platform to participate in the four experiments of the current study shown in Table 1. All participants were monolingual English-speaking individuals currently residing in the United States. Prolific’s “balanced sample” option was chosen to distribute the study to male and female participants evenly. To decide the proper sample size for this study we conducted an *a priori* power analysis utilizing G⋆ Power 3.1 Faul et al. (2007) with the following input parameters: Effect size f = 0.25, *α* error probability = 0.05, and Power 1-*β* error probability) = 0.90. The above analysis suggested a total sample size of 46 participants would be suitable for each experiment. All participants were screened by Prolific to have normal hearing. All participants were required to use reliable headphones during the experiment. 10 participants’ data were excluded from the analysis due to failing the attention checks.

**TABLE 1:** Conducted experiments.

Experiment	N
Standard Monolingual *versus* Standard Bilingual	44
Standard Bilingual *versus* Expanded Bilingual	49
Standard Monolingual *versus* Expanded Bilingual	41
Expanded Monolingual *versus* Expanded Bilingual	48

### Material

For the sentence recognition task, 20 sentences were randomly chosen from the standardized “test of speech intelligibility in noise using sentence material” developed by Kalikow and Stevens (Kalikow et al., 1977) for the English language. All the sentences were thoroughly investigated by Kalikow and Stevens for the effect of phonetic and prosodic factors. Furthermore, the effect of learning was controlled so that “when successive test forms are presented, or even within a given test form” the effect of learning on the performance of subjects did not exceed 1.2 percent. Sentences were chosen from the low predictable form so that the last word could not be inferred from the context of the sentence. As all the original sentences had one clause, to be able to add pause within the sentences, a second clause was added to all the sentences which always was a verb + “ing” using a connecting word (while, as, since, when, by). The verbs were controlled to not have any connections with the first clause in terms of meaning (see the sentences list in the appendix).

Final sentences were shuffled and randomly assigned to 4 forms of 5 sentences each. The participant’s task was to recognize the last word of each clause from 4 options, resulting in 2 questions for each sentence (10 questions for each sentence form). Two reading passages with the same difficulty level were chosen from the book “Master the TOEFL” for the speech comprehension task. Each passage was summarized to be equal in length (each containing 240 words). Nine multiple-choice questions were asked about each passage that included both main ideas and memory of details. All the prepared text materials were translated to Japanese by an experienced Japanese translator to be used for the background language. The text material in both languages was then converted to audio format using the Murf Studio’s AI voice generator. For the target language “English,” the voice of Ava, a young female American adult character was used. For the background language “Japanese,” the voice of Sakura, a young female Japanese adult character was used. The sound characteristics of each condition are shown in Table 2.

**TABLE 2:** Sound characteristics.

Sound	WPM	SPS	Pitch (Hz)	LTAS (dB)
English Original	188	4.3	220	28
English Time-expanded	154	3.4	220	28
Japanese Original	210	6.5	184	27.8
Japanese Time-expanded	171	4.7	184	27.8

The loudness of all voices was normalized to the standard -23 Loudness Unit Full Scale (LUFS) using the Audacity software. The Long-Term Average Spectrum (LTAS) of all the clean speech excerpts was controlled using Praat software to have the same overall long-term spectrum. For the standard monolingual and bilingual conditions, no modification was applied to the generated voices. The standard speed and pause duration implemented by the AI voice generator was kept intact. For the time-treated monolingual and bilingual conditions, the speed of the voice generator was set to 0.6 of the standard speed. In addition, 1 s of pause was added between each sentence (12 pauses in total), and 0.6 s of pause was added between the clauses of each sentence (8 pauses in total). The sounds were mixed using the open-source software Audacity with a 3dBs gain for the target language “English.” The experiments were designed and conducted using the Psychopy software, a GUI Python-based platform for psychological and neuroscientific experiments.

### Procedure

**FIGURE 2:**
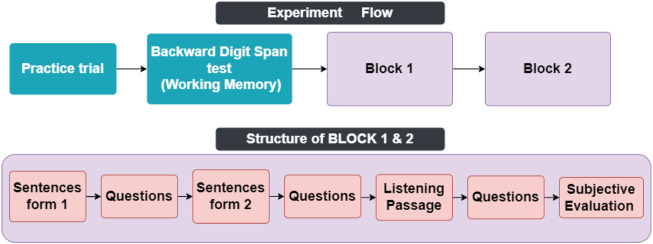
Task flow of each experiment. **(A)** shows the task flow of the experiment, and **(B)** shows the content of each block.

In the beginning, participants started the experiment by reading a written consent form describing the flow of the experiment and were told they are free to leave the experiment at any time by double-pressing the escape button. Then they were instructed how to respond to questions. The experiment consisted of 3 main parts shown in Figure 2. A Backward Digit Span test at the beginning served both as an attention check and exclusion criteria for effortless responses. A single practice trial of the Backward Digit Span test with 3 digits was played for them before the main test, followed by displaying the correct answer of the trial with two intentions. First, to make sure that participants have understood the instructions on how to perform the tasks, and secondly so that participants can adjust the computer volume to their liking so that they can hear every detail of the voice clearly.

The backward digit span test is a well-documented measure to estimate the working memory capacity of subjects (Hilbert et al., 2015). They went through 4 trials in the main test in each of them a list of digits (4, 5, 6, and 7 digits respectively) was played for the subject, and they were asked to write them in the opposite order right after the list finished being played and a star appeared at the middle of the screen in the location [0, 0]. Only responses where all the digits were remembered in the exact order were counted as the correct answer. 10 subjects with a score of 0 in the working memory task were excluded from the data analysis.Then participants proceeded to the main experiment which consisted of two blocks with identical structures. In each block, first, two lists of five sentences were played for them. After each list, they were asked to answer 10 multi-option questions recognizing the last word of each clause of the sentences. Afterward, they listened to a short lecture and were asked to listen carefully as some questions about the content of it will be asked later. After the lecture finished, they answered 9 multi-option questions about the gist of the lecture and its details. The order of the blocks and the order of the sentence lists were counterbalanced so each block, and each sentence list had an equal chance of being played at any given sequence during the task. Each block was followed by a subjective evaluation question where subjects were asked to rate the ease of listening and likability of the recent block on a Likert scale from 1 to 6 where 1 means very easy and 6 means very difficult.

## Results

### Experiment 1

**FIGURE 3:**
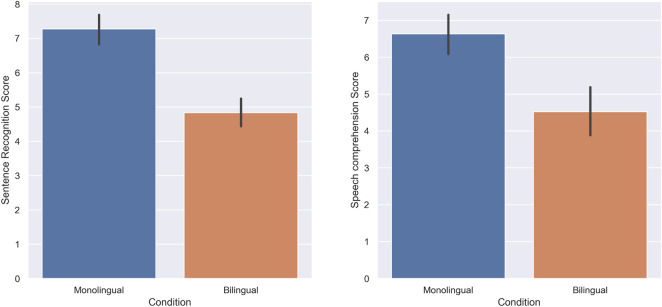
Experiment 1: Standard Monolingual and Standard Bilingual. The score of subjects in the Sentence recognition **(A)** and Speech comprehension **(B)** tasks.

In the first experiment, the score of subjects in standard monolingual (English only) *versus* standard bilingual (unmodified Japanese language as the background language) conditions was tested. Shapiro-Wilk test of normality revealed that the score of subjects departs significantly from normality in both sentence recognition and speech comprehension tasks in both monolingual (W = 0.86, *p*-value = 0.00, W = 0.92, *p*-value = 0.00), and bilingual groups (W = 0.96, *p*-value = 0.02, W = 0.94, *p*-value = 0.04) respectively. As a result, Wilcoxon signed-rank tests were used to analyze the score of subjects in the sentence recognition, and speech comprehension tasks in monolingual and bilingual settings.The score of subjects in the sentence recognition task was higher in the monolingual condition (M = 7.27, SD = 2.08) compared to the bilingual condition (M = 4.82, SD = 1.95); there was a statistically significant decrease when the speech was presented bilingually (W = 232.5, *p* = 0.00, r = 0.84). The speech comprehension score of subjects was also higher in the monolingual condition (M = 6.63, SD = 1.87) compared to the bilingual condition (M = 4.52, SD = 2.26); there was a statistically significant decrease when the speech was presented bilingually (W = 100.5, *p* = 0.00, r = 0.75). Figure 3 illustrates the score of the subjects in both tasks respectively.

### Experiment 2

In the second experiment, the performance of subjects in standard bilingual (English and Japanese with no modification) *versus* time-expanded bilingual (English and Japanese both expanded in time) conditions were tested. Based on the Shapiro-Wilk test of normality the score of subjects did not follow a normal distribution in both sentence recognition, and speech comprehension tasks in standard bilingual (W = 0.96, *p*-value = 0.00, W = 0.94, *p*-value = 0.00), and expanded bilingual groups (W = 0.93, *p*-value = 0.00, W = 0.94, *p*-value = 0.00) respectively. Hence, Wilcoxon signed-rank tests were used to analyze the score of subjects in the sentence recognition, and speech comprehension tasks.

**FIGURE 4:**
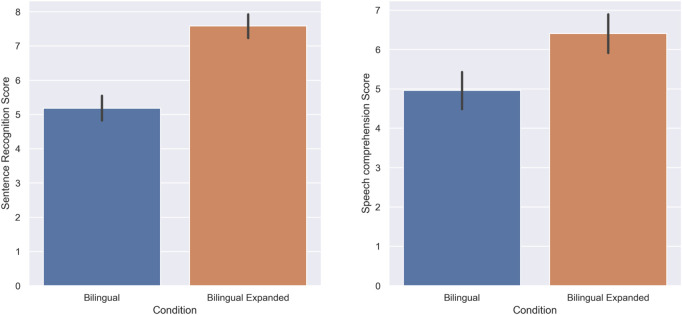
Experiment 2: Standard Bilingual and Expanded Bilingual. The score of subjects in the Sentence recognition **(A)** and Speech comprehension **(B)** tasks.

The score of subjects in the sentence recognition task was higher in the expanded bilingual condition (M = 7.58, SD = 1.72) compared to the standard bilingual condition (M = 5.18, SD = 1.93); there was a statistically significant increase when the speech was presented in an expanded bilingual manner (W = 196, *p* = 0.00, r = 0.89). Furthermore, the speech comprehension score of subjects was also higher in the expanded bilingual condition (M = 6.4, SD = 1.77) compared to the standard bilingual condition (M = 4.95, SD = 1.75); there was a statistically significant increase when the bilingual speech was presented in an expanded manner (W = 116, *p* = 0.00, r = 0.74). Figure 4 shows the score of subjects in this experiment.

### Experiment 3

In the third experiment, the performance of subjects in standard monolingual (English only) *versus* time-expanded bilingual conditions was tested. Shapiro-Wilk test of normality showed that the score of subjects departs significantly from normality in both sentence recognition and speech comprehension tasks in both monolingual (W = 0.91, *p*-value = 0.00, W = 0.94, *p*-value = 0.00), and expanded bilingual conditions (W = 0.87, *p*-value = 0.00, W = 0.9, *p*-value = 0.00). As a result, Wilcoxon signed-rank tests were used to compare the score of subjects in the sentence recognition and speech comprehension tasks in the monolingual and bilingual settings respectively.

**FIGURE 5:**
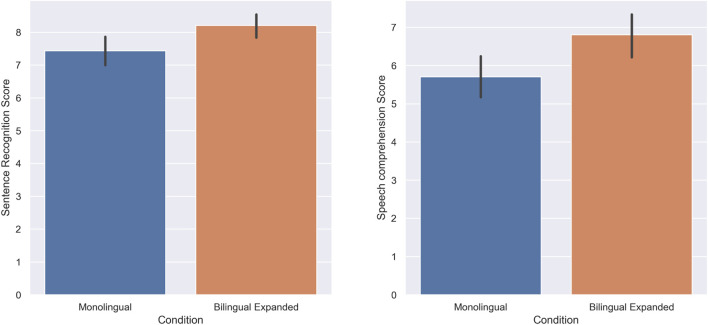
Experiment 3: Standard Monolingual and Expanded Bilingual. The score of subjects in the sentence recognition **(A)** and Speech comprehension **(B)** task.

The score of subjects in the sentence recognition task was higher in the expanded bilingual condition (M = 8.2, SD = 1.69) compared to the standard monolingual condition (M = 7.43, SD = 2.01); there was a statistically significant increase when the speech was presented in an expanded bilingual manner (W = 612.5, *p* = 0.00, r = 0.41). Furthermore, the score of subjects in the speech comprehension task was also higher in the expanded bilingual condition (M = 6.8, SD = 1.86) compared to the standard monolingual condition (M = 5.7, SD = 1.79); there was a statistically significant increase in the speech comprehension of subjects when the speech was presented in an expanded bilingual condition (W = 104, *p* = 0.00, r = 0.58). Figure 5 shows the score of the subjects in the sentence recognition task and speech comprehension task respectively.

### Experiment 4

In the fourth experiment, the score of subjects in time-expanded monolingual (Expanded English only) *versus* time-expanded bilingual (English and Japanese both expanded) conditions was tested. As the score of subjects departs significantly from normality in both sentence recognition and speech comprehension tasks in expanded monolingual (W = 0.96, *p*-value = 0.00, W = 0.93, *p*-value = 0.00), and expanded bilingual conditions (W = 0.91, *p*-value = 0.00, W = 0.9, *p*-value = 0.00), Wilcoxon signed-rank tests were used to compare the score of subjects in sentence recognition and speech comprehension tasks in the monolingual and bilingual settings respectively.

**FIGURE 6:**
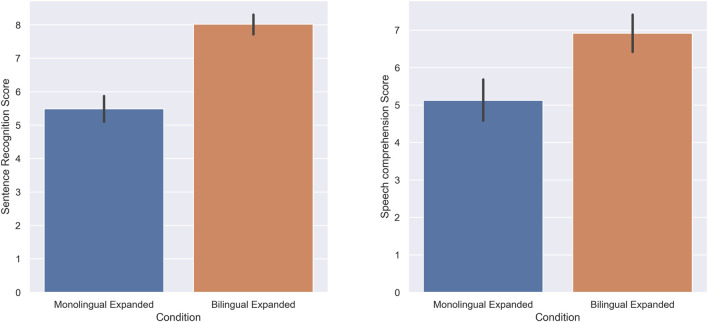
Experiment 4: Expanded Monolingual and Expanded Bilingual. The score of subjects in the Sentence recognition **(A)** and Speech comprehension **(B)** tasks.

The score of subjects in the sentence recognition task was higher in the expanded bilingual condition (M = 8.02, SD = 1.56) compared to the expanded monolingual condition (M = 5.48, SD = 2.03); there was a statistically significant increase when the speech was presented in an expanded bilingual manner (W = 79, *p* = 0.00, r = 0.95). Furthermore, the score of subjects in the speech comprehension task was also higher in the expanded bilingual condition (M = 6.91, SD = 1.78) compared to the expanded monolingual condition (M = 5.12, SD = 1.9); there was a statistically significant increase in the speech comprehension of subjects when the speech was presented in an expanded bilingual condition (W = 82.5, *p* = 0.00, r = 0.79). Figure 6 shows the score of the subjects in the sentence recognition task and speech comprehension task respectively.

## Discussion

In this line of study, we first tried to see if we can repeat the previous findings regarding the negative effect of the presence of background speech on the comprehension of the target language in a competing talker scenario. This step would be crucial to justify the necessity of further research on how to reduce or eliminate this adverse effect in the case of a simultaneously speaking bilingual robot. For this aim, we used two cognitive tasks with different difficulty levels. Sentence recognition, which relies mostly on the immediate recognition of the presented material and hence, is considered a lower-level task that does not impose a high cognitive load on the subject while still shown in previous works to be sensitive to subtle changes in the sound qualities (Hanley and Morris, 1987). Speech comprehension, on the other hand, is considered more cognitively demanding as it requires subjects to follow the story while keeping the key points in mind for a longer time.

In the first experiment, we could confirm the previous findings indicating that the score of subjects in both tasks significantly dropped in presence of a second language even though all the subjects were monolingual English-speaking individuals who were requested to listen to the English speech and did not have any knowledge of the Japanese language (the background language). This is in accordance with the previous body of evidence and suggests that participants find it significantly more difficult to stay focused on the presented content when there is an irrelevant speech present in the background (Oswald et al., 2000; Boman, 2004; Har-shai Yahav and Zion Golumbic, 2021).

Both the results of previous research as well as what we observed in the first experiment of this study indicate that if the presentation of a piece of information in more than one language in a simultaneous manner is in mind, special measures should be taken to ensure intact comprehension and comfortable interaction. There are solid works in favor of a positive effect of time-expansion in speech for the speech comprehension and memory performance of subjects both in older adults with hearing impairment (DiDonato and Surprenant, 2015), as well as younger adults with learning disabilities (Bradlow et al., 2003). Hence, we proceeded with the second experiment where we tested the effectiveness of using time expansion in both the target and background languages as a technique to compensate for the negative effect of the background noise in a simultaneously speaking bilingual robot.

The result of our second experiment indicates that when the speech in both languages is time-expanded, the score of subjects in both tasks significantly increases in comparison with the standard-speed bilingual speech where neither the target speech nor the background one is modified in terms of the time expansion. The result of this experiment suggests that an expansion in the speech time and pause duration can compensate for the negative effect of strong background noise in a simultaneously presented bilingual speech. This can be backed up by works that suggest in an adverse listening condition, using slower speech can improve intelligibility (Haake et al., 2014; Tanaka et al., 2011).

To make matters clear on how the time-expanded bilingual speech can compare with the standard-speed monolingual speech, another experiment was conducted. In this experiment, the performance of subjects in the standard monolingual speech as a baseline was compared with that of a time-expanded bilingual speech. Interestingly, however, the time-expanded bilingual speech outperformed standard monolingual speech in both sentence recognition, and speech comprehension tasks. Based on previous works, the presence of background noise in general results in reduced comprehension by increasing the perceptual effort of listening (Peelle, 2018). This negative effect is explained by researchers as when the to-be-attended and to-be-ignored speeches compete for cognitive resources and this results in reduced comprehension of the presented content (Har-shai Yahav and Zion Golumbic, 2021).

There are a few exceptions, however, like a study that shows children with attention deficit hyperactivity (ADHD) benefit from the presence of a moderate level of background noise (Soderlund et al., 2007). The observed noise-induced improvement in the mentioned study has been justified by a model called “Moderate Brain Arousal.” In this model, it is suggested that moderate levels of noise can result in an increased release of dopamine in the brain which as a result improves differentiation between the target signal and the background noise in ADHD individuals who otherwise suffer from not being able to sustain their attention on the target signal for a long time. Non-etheless, due to fundamental differences between the work by Soderlund et al., and the current study due to the fact that they used white noise as their background noise which is a continuous noise with completely different properties in comparison with the human speech which was used in our study as the background noise, in addition to their participants being diagnosed with ADHD, obtained pattern of results in that experiment is not easy to generalize to the current study. But in general, what can be inferred from the results we obtained in this experiment is that under certain conditions, not only the background noise does not disturb speech processing, but it may be able to improve it, which is a counter-intuitive notion at the first glance. This notion is supported by participant feedback we received like: “Somehow when there are two languages, I can remember everything!” which We only got in the expanded bilingual condition but not in the standard-speed bilingual condition.

However, still, one can speculate that the observed improvement in the time-expanded bilingual speech in comparison with standard-speed monolingual speech might be merely due to the time expansion in the expanded bilingual speech. The logic, in this case, would be that time expansion provides more time for the participant to process the incoming speech signal, or as (Picheny et al., 1986; Tanaka et al., 2011) put it, more cognitive resources would be provided for the subjects of the expanded condition. As a result, one can expect that the time-expanded monolingual speech would result in even higher performance in the participants. To make this matter clear another experiment was conducted that compared time-expanded monolingual speech with time-expanded bilingual speech so that the effect of time expansion is kept constant in both conditions and therefore, the effect of the presence of an expanded background speech is highlighted.

As expected, the score of subjects in the time-expanded bilingual speech was shown to still be significantly higher than in time-expanded monolingual speech. This suggests that the observed improvement in the time-expanded bilingual speech cannot be merely explained away by the time expansion, as then the same effect must have been observed in the expanded monolingual speech as well. The results obtained in this study suggest that the observed facilitating effect is induced in the expanded bilingual condition is induced by the combination of both factors, namely time expansion and presence of an expanded background speech. Previous works that have shown the positive effect of speech expansion mostly have implemented this technique to compensate for a secondary cause of disturbance either caused by hearing impairments due to aging, or a secondary source of background noise Bradlow et al. (2003); DiDonato and Surprenant (2015); Tanaka et al. (2011). The result of this study suggests that when there is no such factor that could disturb speech comprehension, and subjects enjoy a normal hearing, expanding the speech in time not only does not seem to improve the performance of subjects, but can harm it by creating a boring and under-stimulating listening atmosphere for the subjects. The feedback authors received from one of the subjects of the expanded monolingual condition who referred to this condition as “boring” can be another indication that the observed drop in the score of subjects in the expanded monolingual condition might happen due to under-stimulation in the participants listening to this type of speech. This finding can be interpreted by the Yerkes-Dodson law which implies that “the quality of performance in any task is an inverted U-shaped function of arousal” Yerkes Dodson (1908). In this model both under-stimulation and over-stimulation can lead to a deterioration in the performance of subjects in a given task.

Based on what we observed in this study, it seems like there are certain situations where the presence of the background speech can have a positive effect on how much information participants can remember from the target speech as long as both the target language and the background language are time-expanded. The observed improvement in the score of subjects in an expanded bilingual speech is suggested to be due to the collective effect of speech expansion, and the presence of an expanded speech in the background. The positive effect of speech expansion is suggested in previous studies to be due to the extra cognitive resources it provides for the subject to process what they hear by giving them more time while listening Tanaka et al. (2011). On the other hand, regarding the direct relationship between increased arousal, and increased attention in the existing literatureKahneman (1973), it is suggested in this study that the presence of an expanded background speech in the expanded bilingual condition may provide an optimal level of arousal while listening, which in return results in an increased attention to what is being said, while the speech expansion gives subjects enough cognitive resources to process what they are hearing.

One of the limitations of the current work is that the observed results are obtained only from English-speaking participants. As the simultaneously speaking bilingual robot is intended to talk to two people simultaneously in different languages, a future experiment will consider the same effect in people who speak other languages as well. Another limitation of the current work is that it does not investigate the effect of the embodiment of the talking robot on subjects’ performance in a simultaneously speaking bilingual robot. So far, our experiments have tried to cover the paralinguistic factors involved in speech comprehension in noise, however, more parameters can influence the cognitive, and psychological experience of users working with a simultaneously speaking bilingual robot. Non-verbal behaviors that can affect our understanding and impression of the talker and their speech content can be divided into three main domains (Lewis, 1998). In future works, we will try to understand the effect of these parameters namely kinemics which considers the effect of factors like gaze behavior, gestures, and expressive behaviors, as well as proxemics which evaluates factors like conversational distance and body impressions. However, the current finding on the positive effect of speech expansion on speech comprehension in a simultaneously presented bilingual speech can be used in its current form in public address systems (PAS) where the announcement of a piece of information in two languages in a public space is intended. In such a scenario as the results of this study suggests, presenting both languages at the same time in an expanded way not only does not harm the speech intelligibility of the audience of such an announcement, but also can improve their memory of the presented content, and save time conveying urgent announcements in more than one language.

## Data Availability

The raw data supporting the conclusion of this article will be made available by the authors, without undue reservation.
